# Broad Medical Uncertainty and the ethical obligation for openness

**DOI:** 10.1007/s11229-022-03666-2

**Published:** 2022-04-10

**Authors:** Rebecca C. H. Brown, Mícheál de Barra, Brian D. Earp

**Affiliations:** 1 Oxford Uehiro Centre for Practical Ethics, University of Oxford, Oxford, UK; 2 Centre for Culture and Evolution, Brunel University London, London, UK

**Keywords:** Medicine, Ethics, Evidence based medicine, Science communication, Trust

## Abstract

This paper argues that there exists a collective epistemic state of ‘Broad Medical Uncertainty’ (BMU) regarding the effectiveness of many medical interventions. We outline the features of BMU, and describe some of the main contributing factors. These include flaws in medical research methodologies, bias in publication practices, financial and other conflicts of interest, and features of how evidence is translated into practice. These result in a significant degree of uncertainty regarding the effectiveness of many medical treatments and unduly optimistic beliefs about the benefit/harm profiles of such treatments. We argue for an ethical presumption in favour of openness regarding BMU as part of a ‘Corrective Response’. We then consider some objections to this position (the ‘Anti-Corrective Response’), including concerns that public honesty about flaws in medical research could undermine trust in healthcare institutions. We suggest that, as it stands, the Anti-Corrective Response is unconvincing.

Persistent and widespread deficits in medical research create uncertainty about the effectiveness of many treatments. Meta-research that synthesises and scrutinises empirical evidence suggests that problems in the conduct and analysis of scientific studies may produce many misleading results, including a worryingly large proportion of false positives. Work from medical researchers, philosophers of science, methodologists, and others suggests that uncertainty about the exact effectiveness of many medical treatments should be high, and that the structures and incentives operating in medicine facilitate overly^1^ optimistic estimates of treatment value.

In this paper we articulate an epistemic position called Broad Medical Uncertainty (BMU) and explore the ethical consequences of its recognition. BMU results from a lack of adequate evidence of treatment effectiveness in general as well as the stochastic nature of treatment effects on different individuals. A position of BMU is warranted because currently employed estimates of the value of treatments are likely to deviate meaningfully from the true expected value for many, and perhaps a majority, of medical treatments. This results from systematic problems in the collection, aggregation, dissemination and translation of medical research. In Part I we describe BMU and the features of medical research and practice which contribute to BMU and systematically bias in favour of over-optimism regarding treatment effects. In Part II we consider the ethical implications of BMU, arguing that, in the first instance, there is an ethical presumption in favour of openness (e.g. among scientists and healthcare professionals in their dealings with the public) regarding BMU. We address some possible objections to this claim.

## Part I

### Medical Nihilism, medical conservatism, and broad medical uncertainty

In his 2018 book, *Medical Nihilism*, Jacob Stegenga argues that “We should have little confidence in the effectiveness of medical interventions.” (Stegenga, 2018, p. 168) Such a provocative claim might, he acknowledges, seem unreasonable at first: most of us can think of numerous examples of medical interventions improving and prolonging our own and others’ healthy lives. Stegenga lists some important advances in medicine, including the provision of insulin to type I diabetics, antibiotics for infections, and inhalers for people with asthma. We might also note the success of treatments for diseases like HIV/AIDS and some forms of cancer which, thanks to genuine advances in medical understanding and intervention, have gone from being invariably fatal illnesses to being curable or turned into chronic conditions that the patient can live with for many years. Indeed, so obvious is the importance and effectiveness of some areas of medicine that listing examples of valuable treatments seems redundant. Hence one may be inclined to associate Stegenga’s criticisms with those made by fringe groups such as anti-vaccination activists and naturopaths.

Leading medical and scientific authorities have, however, repeatedly made similar claims over the past century. Douglas Altman, then statistical consultant for the *BMJ*, argued in 1994 that “huge sums of money are spent annually on research that is seriously flawed through the use of inappropriate designs, unrepresentative samples, small samples, incorrect methods of analysis, and faulty interpretation.” (Altman, 1994) The situation was, he argued, a “scandal”. A 2018 essay in the *BMJ* argued that many of the fundamental problems identified by Altman continue to undermine cumulative progress in medicine: the scandal persists (Glasziou & Chalmers, 2018). John Ioannidis, a medical doctor and leading figure in meta-research, has detailed the flaws in medical and social science research methods, publication practices and treatment of patients, and has been outspoken regarding the need for reform in empirical research (Ioannidis et al., 2017; Ioannidis, 2005, 2016b, 2018). In their 2015 book, *Ending Medical Reversal*, Vinay Prasad and Adam Cifu highlight the frequency with which accepted medical treatments are subsequently found to be less effective or more harmful than previous (less intrusive) interventions, and argue for changes to medical school curricula and the research and approval processes for medical interventions (Prasad & Cifu, 2015). Margaret McCartney, a GP, broadcaster and frequent contributor to debates on evidence-based medicine, has described how the promotion of interventions such as screening programs cause under-appreciated harms and produce little benefit (McCartney, 2012). In response to these epistemic problems, John Mandrola and colleagues have outlined a position which they describe as ‘medical conservatism’ (Mandrola et al., 2019, p. 900) which “recognizes that many developments promoted as medical advances offer, at best, marginal benefits.”

These and other critiques of medical research and practice detail numerous instances where medical interventions appear to be less effective than is generally assumed. The critiques propose mechanisms that bring this overestimation about, and suggest why ineffective (and harmful) treatments persist in being used. Inaccuracy in beliefs about treatment effectiveness that result from flawed methods of medical research are exacerbated by numerous ‘pollutants’ in our epistemic environment (Levy, 2018), including the growth of ‘predatory’ journals with low publication standards (and high publication fees) that can be hard to distinguish from better quality journals. The result is a collective epistemic position we call ‘Broad Medical Uncertainty’ (BMU). In the next section we summarise some of the processes that lead to BMU, whilst in the remainder of this section we describe in more detail what BMU involves.

Broad Medical Uncertainty refers to a cognitive state comparable to Rumsfeld’s ‘unknown unknowns’ (Graham, 2014). Whilst information about medical treatments’ effectiveness is probabilistic, creating first-order uncertainty about whether any given treatment is likely to be effective for a particular individual, this does not suffice to create BMU. It is uncertainty *about* this uncertainty (further fueled by epistemic pollutants) that leads to BMU. In other words, systemic problems in how data are collected and in how inferences are drawn from these data means that we should expect the true effects of treatment to be outside the range expected by doctors in many if not most cases; but we cannot be confident about whether this is true in any particular case, or if it is true, how inaccurate the estimate is.^2^ In other words, which treatments and what percentage of treatments are misestimated (and by how much) is itself a subject of uncertainty; and we will later argue that the aforementioned epistemic problems are sufficiently common to justify the assertion of BMU. To better illustrate what we mean by BMU it may be helpful to first describe and distinguish the concept from what we might call ‘Narrow Medical Uncertainty’ (NMU).

Imagine a treatment, T, that has been subject to several clinical trials, the aggregated results of which suggest it is effective about 50% of the time. Across trials, the harms and benefits of the intervention are broadly similar (e.g., 45–55% of patients experience a net benefit along some measured dimension) despite variation in the patient samples used and in the specifics of the treatment implementation, just as populations of patients and implementation vary in the real world. Let us stipulate that the trials are unbiased and not subject to fraud. Hence, while each future patient is uncertain about whether they themselves will benefit from the treatment, they can have justified confidence that the trial results will inform them about the probabilities of different outcomes. In relation to T, we (including patients and their medical teams) are in a position of *narrow medical uncertainty.* We can reasonably make an expected value calculation about undergoing the treatment, perhaps with the assistance of a shared decision-making tool.^3^

Now consider treatment T* which has also been subject to several clinical trials, the aggregated results of which suggest about 50% of patients experience a net benefit. However, it is unclear if the health outcomes and statistical analyses reported in these trials were chosen a priori for their rigour and clinical relevance or if they were chosen based on the results they produce. It is also unclear if additional trials have been performed and not reported. Moreover each of the trials suggests that a very different proportion of patients benefit (30% to 70%). It is unclear what explains this variation, but the differences in how the treatment was implemented, which samples were studied, and the measurement of harm and benefit are all plausible explanations. Data from the trials are unavailable for scrutiny and their integrity is not known. A patient considering this treatment would be unwise to predict a 0.5 chance of benefit with any confidence, but it is unclear how they should correct for these epistemic problems. T* is the sort of treatment that leads to *broad medical uncertainty.*

We can describe the uncertainty related to medical interventions as being *epistemic* or *aleatory* (or some combination) (Fox & Ülkümen, 2011; van der Bles et al., 2019). Epistemic uncertainty is associated with a lack of knowledge, for instance, uncertainty about who won the football match yesterday. It can be contrasted with aleatory uncertainty which emerges from inherent indeterminacy in the world, for instance, predicting who will win the football match tomorrow. Epistemic uncertainty is commonly reflected in statements about one’s confidence in a particular claim (e.g. I’m 90% confident that France is bigger than Spain) and can be resolved through additional information or expertise. Aleatory uncertainty is attributed to stochastic behaviour, and cannot be resolved through further knowledge or expertise, though it can be better quantified (flipping a coin hundreds of times will establish, with little room for error, how often it lands heads).^4^ When all epistemic uncertainty has been resolved (e.g. we know that the coin is fair) then the remaining uncertainty is pure aleatory uncertainty, sometimes described as risk (Tversky & Fox, 1995).

Medical interventions will be more or less stable in their effects: some will act consistently across groups, and some will act more randomly. The stability of a treatment’s effects will influence how much aleatory uncertainty is associated with its use. We will also have more or less information about a treatment, including its causal mechanism(s), evidence regarding how it performs in research trials (efficacy), and evidence regarding how it performs in the real world (effectiveness). Good quality evidence will reduce epistemic uncertainty, whilst an absence of evidence, or evidence known to be of poor quality, will increase it. Note that if we have poor quality evidence but do not realise it is of poor quality, it will *not* increase epistemic uncertainty. This is because epistemic uncertainty is subjective: it reflects how confident one *is* regarding a claim, rather than how confident one *should be* about that claim. Poor quality evidence believed to be high quality will make one *feel* one knows more about treatment effectiveness, and hence reduce epistemic uncertainty, even though one is mistaken.

Uncertainty about medical treatments results from both aleatory and epistemic uncertainty. Aleatory uncertainty arises due to the inconsistent effects of treatments on different individuals within a population. Medical research involves chipping away at epistemic uncertainty through evidence collection, and better understanding the parameters of aleatory uncertainty. If this is successful, then we will be in the position of NMU, as with the case of treatment T. If, however, our methods of collecting evidence and using it to judge the effectiveness of treatments are often flawed or poorly executed, or if there is significant inexplicable variation in the effects of treatments, then we will be in a position of BMU.

We argue that we are in the latter, BMU position. This means that the parameters of aleatory uncertainty are typically poorly defined, and that we should have relatively little confidence in our predictions about the effectiveness of many medical treatments. This does not result from *all* medical research being flawed, but from there being sufficient polluting factors in the evidence base (and wider epistemic environment), and it being sufficiently hard to judge when these are present, to cast doubt on the effectiveness estimates of many treatments.

Our claim about the extent of BMU here is somewhat vague. This is, however, partly the point: in the next section we describe some of the factors which make estimates of interventions’ effectiveness unreliable, and which tend to exaggerate their benefits. Yet it is difficult to know the extent to which these factors *in fact* bias estimates of effectiveness (and uncertainty regarding effectiveness) in a given case. Whilst some sources of uncertainty might be better understood by closely evaluating the methods used in a trial or seeking out unpublished research, others are harder to detect or to estimate their impact (consider, for example, research fraud which is by nature covert).

Suppose there were an oracle we could consult to ascertain the true effects of a treatment. If medical uncertainty is unexpectedly broad, this oracle would frequently surprise us since the true treatment effects would be outside our range of expectations. We don’t have an oracle, but we do have follow-up studies that attempt to rigorously reexamine new and presumably superior treatments that have become established in clinical practice. Prasad et al. (2013) reviewed 363 papers reexamining such new practices published in the *New England Journal of Medicine* over a ten year period. 40% of the papers found that, contrary to expectation, the newly introduced practice was no better than pre-existing practices, and 22% were inconclusive.

Unpleasant surprises like this will be less common when the epistemic tools typically employed by medical researchers are well matched to the challenges of drawing inferences about treatment value. Yet another study of evidence supporting primary care physicians’ decision making found that 33% of recommendations regarding treatment were based on “consensus, usual practice, opinion, disease-oriented evidence or case series”, epistemic tools with a poor track record of generating reliable predictions about future treatment effects (Ebell et al., 2017). Another 39% of recommendations were based on “inconsistent or limited quality evidence” from RCTs and cohort studies. Moreover, when guidelines do use widely respected sources of evidence such as, for example, the Cochrane Systematic Review, the findings are rarely conclusive, with only 2% concluding that the benefits are clearly understood and no further research is needed. In 44% of reviews, the authors could not state whether the intervention was net harmful or net beneficial (Villas Boas et al., 2013). In related scientific fields like psychology that use similar scientific tools, attempts to rigorously reproduce key findings have produced many surprises: about half of replications generated findings outside the expected range (Open Science Collaboration, 2015). We cannot extrapolate directly from these studies in order to quantify, with any reliability, the extent of BMU. At the moment we must accept that we are uncertain about our uncertainty whilst having justified concerns that warranted uncertainty extends to many treatments.

It seems unlikely that BMU is widely recognised amongst patients, medical researchers and doctors, although we lack direct insight into their mental states. Whilst attempts are made to improve the quality of clinical evidence, we are not aware of guidelines/treatment recommendations which explicitly address the tendency for medical research as a whole to overestimate benefits (and underestimate harms) and which factor this in to the cost–benefit calculations and recommendations that are made. Moreover, the use of ineffective treatments [as described by (McCartney, 2012; Prasad & Cifu, 2015; Stegenga, 2018)] suggests that awareness of the tendency for the clinical evidence base to inflate expectations is not widespread. Doctors may also lack the skills required to critically appraise the quality of clinical evidence (Maggio, 2016; Smith et al., 2016). Moreover, if the breadth of uncertainty regarding treatment effectiveness *is* known it is likely that it is rarely communicated to patients. A study of 1057 physician–patient encounters found that uncertainty was only discussed 1% of the time for basic decisions, 6% of the time for intermediate decisions and 17% of the time for complex decisions (Braddock III et al. 1999). There have been calls for better training to address the reluctance or inability of doctors to discuss uncertainty appropriately, and even to recognise its presence, since:

Much of medical teaching, including case-based curricula, is driven by the goal of bringing together a constellation of signs, symptoms, and test results into a unifying solution rather than learning how to manage and communicate uncertainty. (Simpkin & Armstrong, 2019, p. 2588)

### Sources of Broad Medical Uncertainty

#### Problems identifying plausible interventions

Medical innovations sometimes come from basic biology and animal studies and, for a range of reasons, we should expect many of these innovations to be false leads. First, human physiology is complicated. Disease processes are typically causally dense: they are determined by a multitude of factors that interact in complex and sometimes unpredictable ways. The animal species used to elucidate these processes often differ from humans in ways that undermine translation to medicine (Akhtar, 2015; Shanks et al., 2009). Furthermore, statistical and methodological problems in animal studies, and basic biology more generally, inflate the importance of chance findings (Fitts, 2011), leading to a high percentage of non-replicable results (Nosek & Errington, 2017). A recent attempt to replicate 193 influential cancer biology experiments found that none reported sufficient information to replicate the studies in the original publications, and just 50 could be replicated with the assistance of the original authors (Errington et al., 2021a). In these, the average effect size was 85% lower than the original studies (Errington et al., 2021b).

Other medical interventions have their origin in cross-sectional and longitudinal studies of human populations. Although many important advances in public health are derived from such observational studies—the harms of smoking, for example—establishing causal relationships from observational data is notoriously difficult. Observed links between hormone replacement therapy, circulating HDL cholesterol, and cardiovascular disease, for instance, have not found support in randomized controlled trials (Davey Smith & Phillips, 2020).

#### Problems testing plausible interventions

A basic and epidemiological science that generated even a small proportion of beneficial interventions would be valuable if there were reliable ways to identify these interventions. However, a large proportion of randomised controlled trials (RCTs), widely considered to be the ‘gold standard’ for assessing the effects of treatments, are not reliably conducted in ways that ensure an unbiased estimate of the treatment’s value. Moreover, of the many RCTs that are conducted, regardless of quality, half of them have not ultimately been published (Song et al., 2010). Here we outline some of the main problems with RCTs (and clinical research more broadly).

RCTs are techniques for establishing whether or not something helps more than it hurts. But often, researchers primarily attempt to measure the intended ‘help’ outcome, without putting as much care into measuring potential ‘hurt’ outcomes, many of which they may have little ability to anticipate (Stegenga, 2016). Many trials are not powered to reliably measure negative side effects and observed negative outcomes are often omitted from trial reports (Singh & Loke, 2012; A Wahab et al., 2013). One study found that a median of 64% of adverse events recorded in trial documentation do not get mentioned in the published reports (Golder et al., 2016); another found that the proportion of systematic reviews with good harm reporting was 0.56 (Zorzela et al., 2014). Another review of reviews found that reporting of side effects was inconsistent and poor across 800 RCTs (Hodkinson et al., 2013). As Bonell et al. (2015) point out, harms are poorly measured in public health interventions as well as in treatment evaluations. So in many cases, “absence of evidence” really is no evidence of absence.

Meanwhile, the measures that are used for assessing benefits are not always adequately validated. A low-quality questionnaire will yield low-quality data, its being included in an RCT notwithstanding. In other words, merely being embedded within a type of study that, *if well-designed end executed*, may produce good causal evidence for certain claims in certain contexts (Cartwright, 2011), cannot transform a faulty measurement into a good one (Earp, 2016).

Familiar problems with RCTs and other medical studies—even if the design itself is sound, with good measurements of all relevant variables (something that is rarely, if ever, achieved)—include selection bias, p-hacking, outcome switching, and publication bias. These are described in Box 1, along with the measurement problems described above. In short, attempts to study the effectiveness of medical interventions incorporate many ‘researcher degrees of freedom’ or ‘malleability’ (Simmons et al., 2011; Stegenga, 2018, pp. 84–98). These are opportunities for decisions by researchers to influence the outcomes of analysis—selecting what measures to use, statistical tests to apply, results to include and exclude, and so on.

Such flexibility could, in theory, cause underestimation of effectiveness as frequently as overestimation. However, the effect of malleability is not random. The influence of researcher decision-making on what results are generated from medical trials tends towards overestimating effectiveness. For example, when researchers describe the effects of an intervention on an outcome that had not been included in a pre-specified plan, the ‘benefits’ of the intervention are 16% larger than in trials where the plan was followed and this flexibility is not exploited (Chen et al., 2019). Researchers’ ability to exploit this flexibility and inflate benefits may explain why industry funded RCTs tend to generate more positive results (Flacco et al., 2015; Lexchin et al., 2003; Vlad et al., 2007). The pervasiveness of bias and conflicts of interest within medical research are key factors in ensuring that malleability leads to over- rather than under-estimation of medical intervention effectiveness. In Box 1, we detail a range of processes that should undermine our confidence in results stemming from RCTs.

**Table:** **Box 1** Description of some of the methodological factors which contribute to the overestimation of the effectiveness of medical interventions

Selection bias/enrichment strategies	Selection bias results from salient differences between control and experimental groups besides the intervention in a trial, and may result from ‘enrichment strategies’—the intentional inclusion/exclusion of participants from a trial in order to influence the results. Randomisation is intended to mitigate against selection bias, but is not always used appropriately (Pildal et al., 2007; Stegenga, 2016)
Surrogate end points	Surrogate end points may be used as proxies to estimate how effective an intervention is (e.g. the use of HbA1c as a measure of diabetic control; tumour size as a surrogate for cancer survival). Although improvement in surrogate outcomes is often the sole basis for treatment approval and implementation, these surrogates often fail to reliably track the outcomes that we ultimately care about, like survival (Kemp & Prasad, 2017)
Poorly designed instruments	Measures have been developed to assess the effects of interventions on things that we care about—e.g. to see what effect antidepressants have on people with depression. But these measures may distort the picture of the effect of an intervention. Stegenga illustrates this using the Hamilton Depression Rating Scale which scores people on the severity of their depression. According to this scale, if an intervention reduces insomnia but has no effect on the intensity of depression someone is feeling, it may still be recognised as an effective treatment for depression (Stegenga, 2018, pp. 115–117)
P-hacking	Flexibility in choice of statistical analyses, participant inclusion criteria etc. can be exploited to generate statistically significant findings. If a dataset can be plausibly analyzed in numerous different ways, researchers sometimes select the specific analysis that spuriously generates a *p*-value (the probability the data would differ at least as much as observed from a model representing ‘no effect’) below some alpha threshold (an often arbitrary decisional threshold for rejecting the no-effect model). Evidence for p-hacking appears in small industry funded trials (Adda et al., 2020)
Outcome switching	Where researchers specify in a trial protocol that they will measure a particular outcome to judge effectiveness, but after results have been collected and the trial is written up for publication, they report a different outcome instead or in addition (an outcome that typically makes the intervention appear more effective than the originally specified outcome does) (Altman et al., 2017). Studies suggest switching is widespread: 24% of primary outcomes reported in 67 trials in high impact journals in 2015 were switched while just 40% of 192 trials published in 2013 had “clearly defined, prospectively registered outcomes that matched the published outcomes” (Jones et al., 2018; Goldacre et al., 2019)
Passive harm detection	Most data on the harms of interventions comes from passive surveillance and observational studies (contrast this with the careful design of trials to detect even a small benefit of an intervention). This means that many harms go un(der)reported and sometimes ignored
Publication bias	The results of about half of trials have never been reported (Song, Parekh, et al. 2010, Ross, Mulvey et al. 2009). 33% of trials on the EU clinical trials register and 29% of trials on ClinicalTrials.gov contravene requirements to report results within a year (Goldacre, DeVito et al. 2018, DeVito, Bacon et al. 2020). Positive results (those showing an intervention to be effective) are more likely to be published than ‘negative’ results (Song et al., 2010). Positive findings tend to be regarded as more important/higher impact and thus are more appealing to the editors of journals. Further, those funding research (such as pharmaceutical companies) have a greater incentive to publish evidence that their intervention is effective than research which shows it to be ineffective or harmful (DeVito & Goldacre, 2019)
Samples are not representative	Trials are often performed on a set of people who are different from the set of people who end up receiving that treatment. One big difference is prevalence of multi-morbidity (Fortin, 2006; Barnett et al., 2012). This can mean that the effectiveness of the intervention is exaggerated in the research participants relative to the patient group, whilst the harms experienced by participants are likely to be fewer than those experienced by patients. The problem can be exacerbated by shifting definitions of disease—often commercially motivated—which further expands the set of people who may be treated (Moynihan et al., 2019; Stegenga, 2018, pp. 40–53)

#### Problems in evidence synthesis

Systematic review and meta-analysis of multiple RCTs evaluating a treatment are often used to inform policy. These methods of evidence synthesis cannot make reliable estimates of treatment benefits and harms if the trials that they synthesise are flawed (the so-called ‘garbage in, garbage out’ problem). If, for example, the trial authors exploit methodological flexibility to inflate treatment benefits or fail to power a study to detect harmful side effects, the systematic review that summarises this evidence will be unduly optimistic about treatment benefits and harms. Well-conducted systematic reviews can identify some methodological problems in trials: one review of Cochrane reviews found that high quality evidence was available for just 10% of the primary health outcomes examined (Conway et al., 2017). As Roberts et al. (2015) point out, systematic reviews’ emphasis on including *all* relevant trials may be misplaced when so many trials have fundamental methodological flaws. Indeed, findings of systematic reviews of multiple weak trials have in the past been contradicted by a single well-powered and rigorous trial. Like many of the issues outlined here, there are partial solutions. For example, meta-analysis of individual-level data rather than of summary estimates may allow for more accurate estimates of treatment value, and careful screening can be used to identify problematic trials and biased estimates. Our point is that much of the evidence that comes from systematic reviews as they are currently practiced is likely to be biased due to problems with the underlying trial data (Ioannidis, 2016a).

#### Conflicts of interest and perverse incentives

Conflicts of interest can bias the results of clinical trials. They can also influence the actions of patient advocacy groups, systematic reviewers, clinical guideline developers, regulatory agencies and their advising committees, medical educators, textbook authors and medical journals (Stegenga, 2018, p. 161; Moynihan et al., 2019). Conflicts of interest need not undermine researchers’ integrity to be corrosive. Holman and Bruner (2017) point out that industry funding flows selectively towards people that happen to conduct research or hold views that supports funders’ commercial goals (see also (Howick, 2019). In the absence of strong financial support for other research approaches, a consensus around the ‘benefits’ of harmful or low-value treatments can emerge.

Major medical journals play an important role as promoters of biomedical research and have the capacity to draw attention to and add credibility to certain results. However, perverse incentives are common in publishing. Major medical journals are incentivised to publish certain trials because the trial funders purchase tens of thousands of reprints of the articles describing the effects of their treatments: A 2010 study found that 41% of the *Lancet’s* income, and 3% of the *BMJ’s,* came from such reprint sales (Lundh et al., 2010). Other leading journals declined to provide data to this study, but the practice appears widespread. The *New England Journal of Medicine*, for example, sold 900,000 reprints of an article describing the benefits of rofecoxib to its developer Merk before the drug was subsequently withdrawn from the market due to cardiovascular side effects. Publications with large reprint orders are typically pharmaceutical industry funded trials (Handel et al., 2012). Few leading journals regulate the contribution of authors who have strong incentives to minimise harms or maximise benefits in their scientific writing (Lundh et al., 2020). The malign effects of such conflicts of interest has been noted by editors of leading journals including Richard Smith, former editor of the *BMJ* (“medical journals are an extension of the marketing arm of pharmaceutical companies”) (Smith, 2005), Richard Horton, editor of the *Lancet* (“journals have devolved into information laundering operations for the pharmaceutical industry”) (Horton, 2004) and Marcia Agnell, former editor of the *New England Journal of Medicine* (“It is simply no longer possible to believe much of the clinical research that is published”) (Angell, 2009).

Fraud presents a particularly hard to evaluate pollutant in the clinical research literature since, by its very nature, it is covert. There have been a number of high profile cases of fraud relating to medical research, including Paolo Macchiarini, a surgeon at the Karolinska Institute who transplanted artificial tracheas into patients and misrepresented the results in publications, including in the *Lancet*, to suggest the transplants were a success when in fact many of the patients died (Ritchie, 2020). Individual cases of dramatic fraud do not tell us much about the pervasiveness of such practice, however. In the field of microbiology, attempts have been made to evaluate the frequency with which images of western blots in journal publications are altered in problematic ways. Bik and colleagues found dishonest techniques had been used in 3.8% of papers published across forty biology journals (Bik et al., 2016). A meta-analysis and systematic review of survey data found that 1.97% of scientists "admitted to have fabricated, falsified or modified data or results at least once”, whilst 14.12% said they had personal knowledge of a colleague doing so (Fanelli, 2009). Such surveys are unlikely to give an accurate picture of the frequency of fraud in scientific research, but they indicate that it is by no means absent.

#### Problems translating evidence into practice

Finally, additional reasons for BMU arise in the translation of scientific findings into practice. Even unbiased trial results can be ‘spun’ in misleading ways: one study found 23% of 138 anaesthesiology trial abstracts made claims which were not justified by the trial’s results (Kinder et al., 2019). Despite this and the other problems outlined above, RCTs are often the most effective way to quantify the harms and benefits of medical interventions. Studies of influential clinical guidelines, for example, suggest that less than half of recommendations are based on studies capable of providing unbiased estimates of treatment value (Feuerstein et al., 2014; Lee & Vielemeyer, 2011; Venus & Jamrozik, 2020). Moreover, if trials strongly suggest treatments to have no net benefits, it can take years or even decades for them to be withdrawn once they are in broad usage (Montini & Graham, 2015; Niven et al., 2016).

While there are good reasons to think that medical science overestimates the value of treatments, Hoffman et al. (2017) found that doctors’ expectations are still more optimistic, even than the (likely biased) evidence base suggests. A range of factors may drive these beliefs. First, doctors’ judgements of treatment value may be informed by their own observations of patient recovery, which may mistakenly credit interventions with benefits that result from simple regression to the mean or natural healing (Morton & Torgerson, 2005). Word of mouth about treatment effects is strongly biased towards the positive, meaning that people hear more about successes than average outcomes (de Barra et al., 2014; de Barra, 2017). Low statistical literacy levels among doctors and other health care workers may further undermine their ability to translate scientific findings into beneficial patient care (Wegwarth et al., 2012).

### Existing improvement measures

One obvious solution is to tackle BMU directly. Reducing BMU can operate either by using more reliable tools for assessing the effectiveness of medical interventions (e.g. improving trial methodology), or developing and using tools to better quantify uncertainty about medical effectiveness—transform BMU into NMU by gathering better information about the uncertainty in effectiveness for different interventions.

Many of the efforts of those working in Evidence Based Medicine are aimed at this. For instance, the AllTrials initiative campaigns to ensure that all past and present clinical trials register their methods and publish a summary of their results, in order to mitigate the effects of publication bias and outcome switching and open the methods up to scrutiny more generally (AllTrials, 2014). In 2004, the International Committee of Medical Journal Editors announced registration would be a pre-requisite for clinical trial publication. Research funders, including the Wellcome Trust, the Medical Research Council, Cancer Research UK and the Bill and Melinda Gates Foundation have made further commitments that data sharing should be mandated (Kiley et al., 2017).

Such efforts doubtless improve transparency and the appraisability of the clinical research evidence base, but practices like pre-registration come with a number of challenges (Nosek et al., 2018) and many trials register retrospectively (Harriman & Patel, 2016). Further, there has been variable uptake and limited enforcement of registration requirements globally (Viergever & Li, 2015). Even when study protocols are available, discrepancies in pre-specified and reported outcomes are prevalent, and journals are often unwilling to publish corrections: Goldacre and colleagues wrote letters to editors at five high impact journals every time a trial misreported its outcomes over a six week period. Correction letters were published only 40% of the time, and often with long delays (Goldacre et al., 2019). Journal policies may also fail to reflect appropriate standards of transparency and requirements for disclosing conflicts of interest (Cashin et al., 2020). Efforts to promote data-sharing have included the provision of ‘badges’ to acknowledge good practice, but this failed to motivate authors of articles published in *BMJ Open* to share their data (Rowhani-Farid et al., 2020).

These efforts are laudable and there have been significant improvements in the reliability of the evidence base, and we can expect more to come. Whilst we wholeheartedly support these efforts at reform, they have not (and may never) eliminate BMU and for at least some (perhaps many) interventions NMU may be unachievable. Despite the efforts of many committed reformers, much (seemingly warranted) epistemic uncertainty still persists (Greenhalgh et al., 2014). Many currently used treatments pre-date such reforms, and limited uptake and enforcement mean that the problems we describe with the clinical evidence base persist. Moreover, communicative practices downstream of evidence collection add further to over-optimism regarding treatment effectiveness.

## Part II

### An ethical argument for openness about BMU

Above, we have described some of the contributors to BMU and explained why there is significant uncertainty regarding the effectiveness of medical interventions, and why intervention benefits are likely to be overestimated by researchers, physicians and patients. If this picture is accurate, the current situation is troubling. Money is wasted on, and people are harmed by, overtreatment and ineffective interventions.

If we are unable to resolve BMU, at least for the time being, through better evidence and scientific models of disease, then we must manage it via other means. We suggest that there is a prima facie ethical case for greater openness regarding BMU. We take openness to mean something close to transparency: making the processes involved in conducting scientific research and the production of treatment recommendations visible. But, following O’Neil, we adopt the term ‘openness’ to reflect a need to go beyond merely disclosing technical information. ‘Intelligent openness’ requires further efforts to make that information meaningful (The Royal Society, 2012).^5^ Openness also usefully references the move towards ‘open science,’ and indicates the potential to better engage patients with the processes of medical research and treatment decisions (Munafò et al., 2017). Regarding BMU, openness will require active efforts to make people aware of the limitations and uncertainty of the evidence available regarding the effectiveness of many medical interventions, and additional humility when reporting treatment effectiveness and making recommendations. This ethical case is based on epistemic, professional and instrumental reasons.

#### Epistemic

It is epistemically valuable to have a more accurate picture of the world. On most ethical theories it is morally wrong (either defeasibly or non-defeasibly) to deceive agents via lying, misinformation, omission, or other means (Carson, 2010; Saul, 2012). It may also (depending on further contextual factors) be wrong to mislead people, even when this is done unwittingly and unintentionally. People’s inaccurate beliefs about the effectiveness of medical interventions are created and maintained, in part, by the actions of others (including healthcare professionals and medical researchers). Given the availability of the evidence described above, this misleading is foreseeable (even if unintentional), which makes it more likely to constitute wrongful misinforming.

#### Professional

The actions of medical practitioners and others working in the delivery of healthcare are guided by professional codes of conduct. These include ethico-legal requirements to, for instance, respect patient autonomy by ensuring they are provided with sufficient information in a comprehensible form to allow them to make decisions about the medical treatments they receive. The General Medical Council, which produces professional guidelines for British physicians, states doctors must “Be honest and open and act with integrity” (General Medical Council, 2020). Supplying information which is misleading or false (for instance, by failing to acknowledge uncertainty around effect size or likely harms) could constitute a failure of these obligations.

#### Instrumental

There are likely to be other, instrumental gains to attempting to correct people’s over-optimistic expectations of medical treatments. For instance, a greater degree of openness regarding BMU might result in more accurate beliefs about the effectiveness of medical interventions, and further, contribute to better decision making (where ‘better’ equates to improved health outcomes or greater well-being or similar). Although speculative, it seems reasonable to assume (in the absence of contradictory evidence) that people will be better able to make good decisions about their medical treatment if they have an accurate picture of treatment effectiveness.

We hold, therefore, that greater openness is presumptively warranted to correct people’s inaccurate expectations of treatment effectiveness and to draw their attention to BMU. Importantly, this is likely to involve reducing expectations of treatment benefits (and increasing expectations of no or harmful effects), due to the tendency for expectations to skew towards optimism. This could involve changes to the way information about treatment effects is communicated in clinical encounters and public health information.

Health communication can be challenging, particularly where the information is complex and recipients have limited health literacy. It has been shown that healthcare providers typically do not communicate uncertainty, and that barriers to doing so include the belief that uncertainty indicates ignorance or failure, and the fear that communication of uncertainty may have deleterious consequences (Braddock III et al. 1999; Simpkin & Armstrong, 2019). Research into the communication of risk and uncertainty can support effective communication, and has been implemented in some settings (Gigerenzer & Kolpatzik, 2017; Gigerenzer et al., 2007; van der Bles et al., 2019). There is also a significant empirical and theoretical literature on medical decision making, often focused on clinical encounters and the requirements for information exchange to meet the standards of informed consent processes and reflect the ideals of shared decision making (Barry & Edgman-Levitan, 2012; Brock, 1991; Faden & Beauchamp, 1986). There are well-rehearsed challenges to conceptualising and enacting superior clinical decision making, such as what counts as a ‘rational’ choice and how much weight to give to rationality, as well as defining, teaching and implementing shared decision making (Brock & Wartman, 1990; Gigerenzer et al., 2007; Wegwarth et al., 2012; Elwyn et al., 2016).

We call efforts to highlight BMU and correct inaccurate beliefs about medical treatments a ‘Corrective’ response and opposition to such a strategy the ‘Anti-Corrective’ response. Efforts to implement a Corrective Response can draw upon and add to existing discussions of decision making in medicine, highlighting a greater role for acknowledging the uncertainty inherent in medical research when considering different interventions’ (or non-interventions’) merits. Further, it suggests a need to address a lack of health literacy, particularly ‘probability’^6^ or ‘statistical’ literacy, amongst patients, clinicians and policy makers in order to facilitate ethical decision making (Gigerenzer & Gray, 2013; McAllister, 2016). Rather than spend the remainder of this paper discussing how the Corrective Response should be implemented, we will instead consider some objections to such a response.

### The anti-corrective response: against openness regarding BMU

In this section we discuss two plausible reasons for thinking that a Corrective Response to BMU would be inappropriate, unjustified or self-defeating. Of course, a rejection of the argument for BMU would lead to an Anti-Corrective Response. We set this aside, and in the following discussion assume that the case for BMU is compelling.

The Anti-Corrective Response proposes that, even if people overestimate the effectiveness of medical interventions, we ought not to seek to correct this misbelief, since to do so would result in negative externalities such as an overall reduction in health or diminished efficiency in healthcare delivery. A weaker version of this argument would suggest that there is no obligation to adopt a Corrective Response; that although no harm results from the Corrective Response, there is also no (or insufficient) good that results from adopting it. Since we believe that there *are* important ethical reasons for adopting a Corrective Response, a case needs to be made for failing to do so.

The first iteration of the Anti-Corrective Response we consider is the concern that, even though people currently overestimate the effectiveness of medical treatments, they *still* fail to engage with them sufficiently: people delay or avoid entirely visiting a doctor when they have a medical complaint for which they ought to seek treatment; they do not take prescription medications as advised; they adopt unhealthy behaviours, and so on. Informing people that the effectiveness of medications is often less certain or less beneficial than they expect might only encourage this (putatively harmful) avoidance and further reduce the effectiveness of medical interventions. The second iteration of the Anti-Corrective Response we consider argues that revealing the extent to which medical research incorporates systematic bias, flawed methodologies and deep uncertainties could cause a dramatic loss of trust in the institutions of medicine and science more broadly. Recent concerns about the spread of ‘fake news’ and the rejection of expertise have increased anxiety around actions that could further undermine the authority of science and traditionally dominant sources of information (Chou et al., 2018; Petersen et al., 2019). We discuss each of these concerns below.

#### Openness regarding BMU will reduce engagement with medicine

People sometimes don’t seek medical care despite being sick, and those who do seek care often fail to adhere to treatment recommendations (Byrne, 2008; NICE, 2009). For instance, around 50% of patients with chronic illnesses do not take treatments as prescribed (Brown & Bussell, 2011, p. 304). In addition, many fail to act in accordance with public health messaging (about 40% of adults in England fail to meet recommended physical activity guidelines (Scholes & Neave, 2017); about a quarter of adults in Scotland drink at ‘hazardous’ levels [over 14 units a week] (McLean et al., 2018)). Perhaps increased openness about Broad Medical Uncertainty could exacerbate these problems. In response to this concern, we argue that (1) patients’ certainty in the value of a treatment is unlikely to be the main cause of adherence, (2) misleading patients (or allowing them to become misled) in order to enhance adherence is unethical, and (3) in some circumstances, patients *should be* unwilling to adhere to treatment recommendations that have uncertain consequences.

There are various reasons why people under-engage with healthcare. Taber et al (2015) characterise a number of these factors, including: low perceived need for medical care (including the belief that one’s illness will get better on its own); traditional barriers to medical care (such as lack of time, or lack of health insurance); unfavourable evaluations of seeking medical care (including concerns about the quality of care or expectation of negative outcomes, often rooted in genuine past mistreatment of certain groups, e.g., racial minorities; (Dovidio et al., 2008)); and self-ascribed personality traits (such as laziness). A similar range of factors influence treatment adherence, including patient-, physician-, and health system-related factors, such as a patient’s lack of understanding of their disease, lack involvement in the decision-making process, high medication costs, poor physician communication, complex drug regimens, and time constraints on clinical encounters (Brown & Bussell, 2011).

Reduced expectations of medical effectiveness could plausibly exacerbate the effects of such factors, though it is unclear this would be the case. Even if highlighting uncertainty reduced adherence, this would be insufficient to justify maintaining widespread false beliefs about the effectiveness of medical interventions. Moreover, if an explicit decision were made to avoid highlighting BMU in order to encourage engagement with medical treatments, there would be a risk of this deception eventually being revealed. In such an event there would be a risk of even less engagement in the longer term, as the public might (perhaps justifiably) feel that they had been duped. As Levy (2018, p. 138) notes, “When trust is lost, it is difficult to restore, since remedial measures taken by distrusted institutions are likely to be regarded with a jaundiced eye”.

Preferable approaches to tackling harmful avoidance and non-adherence could be targeted towards the upstream causes (e.g. addressing financial barriers). Again, the inflation of expectations in medical treatment—and likely subsequent disappointment—may only lead to poorer engagement and adherence long-term.

Further, healthcare avoidance and treatment non-adherence is only a problem if it would, in fact, be better for individuals to seek healthcare or adhere to treatment. Those who have problems which healthcare is ill-equipped to address *should* avoid healthcare; those whose treatments do not confer valuable advantages or involve high costs (including unpleasant side effects) *should* be non-adherent.

Unfortunately, knowledge of BMU cannot tell patients (or their medical teams) whether or not *in their case* treatment will be all-things-considered worthwhile. It only provides information at the general level, with regards to the tendency for treatment effect sizes to be overestimated (and harms underestimated), and uncertainty downplayed. Given that most patients will have no special expertise regarding their condition, nor the capacity to read and evaluate the clinical evidence base regarding their treatment alternatives, it seems unlikely that knowledge of BMU will put them in a better position to make treatment decisions than they were in previously. That is to say, despite all we have said, perhaps patients should just trust their doctors’ treatment recommendations *anyway*, notwithstanding BMU?

‘Trust your doctor’ will, indeed, be a reasonable heuristic for many patients. The difference that awareness of BMU makes is that it may be prudent for patients to not follow their doctors’ advice in a wider range of cases. Those who are capable of reading and evaluating the clinical evidence base may come to different conclusions from their doctors’ advice or clinical guideline recommendations. Even for those (many) patients incapable of such engagement with the technical literature, BMU suggests it would be rational to be at least somewhat sceptical about the benefit (and harm) estimates. If a treatment lies close to the borderline between ‘good enough to try’ and ‘harmful enough to avoid’, patients might be prudent to select an alternative treatment or choose no treatment, given BMU, even if this goes against a doctor’s recommendation. Such patients should not, by dint of their failure to follow a doctor’s recommendation alone, be considered ‘anti-science’ or conspiracy theorists. Rather, they may be making rational decisions given reasonable concerns about the quality of evidence upon which their treatment recommendations have been made (in conjunction with any value judgments reflecting their personal preferences or priorities, as we discuss next).

It is already recognised that different forms of expertise exist, and that there are varied reasons, including reasonable grounds, for ‘lay’ folk to not defer to ‘expert’ recommendations some of the time (Lengbeyer, 2016; Levy, 2019). This is often justified on the basis that lay folk have personal value-based expertise which scientists (including doctors) lack. In other words, they have greater expertise in their own values. Moreover, doctors are not, by virtue of their training, necessarily better than all of their patients at drawing logical conclusions from complex premises; some patients may understand a doctor’s recommendation, and appreciate the reasons for them, but nevertheless determine that the cited reasons are not sufficient to put much weight on the recommendation. Medical training and practice experience do not always equip doctors with the needed expertise to make good treatment recommendations for a particular patient. Many generalist doctors lack the time (and perhaps skills) to assess the clinical evidence base and may be unwilling or unable to critically evaluate treatment guidelines (Maggio, 2016; Smith et al., 2016).

BMU suggests that patients can be epistemically justified in declining treatment recommendations from their doctors in a wider range of circumstances than healthcare professionals might ideally prefer. This should be of concern to those working in the medical evidence and treatment pipeline, and incentivise renewed engagement with the reform projects of evidence based medicine in an effort to understand and address the uncertainty that can epistemically warrant such decisions.

#### Openness regarding BMU will result in a damaging loss of trust in medicine and science more broadly

For most people, it is difficult to discriminate between scientific claims which are broadly accepted by the majority of experts in a particular area, and those that are widely considered to be mistaken or fraudulent, since non-experts typically lack the subject specific training needed to make such judgements (Collins, 2014; Levy, 2018). For example, most laypeople who assume vaccines to be (sufficiently) safe and effective do so not on the basis of a careful evaluation of the scientific evidence, but out of trust in the medical profession which recommends vaccination, and adherence to the norm within their social group which tends towards vaccinating (Oraby et al., 2014; Ozawa & Stack, 2013).

This suggests that signifiers of trustworthiness are important to engagement with (effective) healthcare, such as (some) vaccination programs. Indications of poor practice, political deception, and conflicts of interest risk undermining people’s inclination to trust traditional experts (Levy, 2019). Some may instead look to apparently ‘truth-speaking’ whistleblowers to the Big Pharma-led vaccination ‘conspiracy’ (Larson et al., 2011; Leask & McIntyre, 2003; Qiu et al., 2016). Again, this reduction in trust in traditional experts can be, to a degree and in certain respects, rational (Levy, 2019). Collins describes how, for an ordinary citizen, it is impossible to make technical judgements about cases where there appear to be conflicting scientific claims being made: ordinary citizens simply do not have the required (‘interactional’) expertise to judge which claims it is reasonable to accept and which ought to be rejected (Collins, 2014, pp. 102–03). But, Collins offers:

Non-specialists may have enough knowledge of a local situation to understand that the normal scientific process is being distorted. To know this does not require specialist knowledge of the science, only the most general knowledge of what kind of thing science is. (Collins, 2014, p. 103).

This practice Collins describes as ‘local discrimination.’ It is not easy, and not all ‘ordinary citizens’ will be capable of it, but local discrimination offers a way of adjudicating between conflicting specialist expertise that has relevance for one’s own behaviour. A greater appreciation of BMU is likely to influence people’s adjudication between different groups who make claims on expertise, potentially reducing trust in traditional medical experts such as authors of papers published in leading journals, people like the UK’s Chief Medical Officer and organisations like the World Health Organization (WHO).

Openness about BMU provides grist for the mill of those who seek to discredit these experts and promote harmful behaviors like refusing a genuinely safe and effective vaccine. For instance, anti-vaccine activists often highlight that pharmaceutical companies have financial conflicts of interest that may lead them to underplay harms and overestimate benefits (Crosby, 2010). The fact that industry-funded trails of drugs and medical devices *are* more likely to show a positive outcome than trials with other sources of funding (Lundh et al., 2017) seems likely to add weight to their arguments. In the context of the challenges caused by the anti-vax movement and the vivid picture it provides of what can happen when scientific orthodoxy is challenged by spurious counter-evidence, caution seems reasonable in considering a Corrective Response to BMU. Highlighting limitations and uncertainties in medical research and treatment might not merely reduce engagement with medicine and adherence to ostensibly safe and effective treatments, but could risk a more serious undermining of trust in healthcare institutions broadly, adding to the armoury of anti-vaxxers and others who promote insufficiently supported claims.

To assess the force of the Anti-Corrective position here, we need to consider (at least) two questions: (i) Would greater openness regarding the methods of medical research and the practices of healthcare institutions—including more accurate representations of uncertainties regarding the effectiveness of medical interventions—serve to undermine trust in medicine and science more broadly? (ii) If so, would the prospect of this loss of trust justify the passive continuation of practices which lead to undue certainty and optimism about the effectiveness of medical interventions, or even active efforts to shield people from a fuller understanding of how medical institutions operate and the current overestimations of treatment effectiveness?

It is difficult to answer (i). We could look to historical examples where poor scientific practice has been revealed, either via whistleblowers or through voluntary reform, and seek to assess the wider effects of such events on public trust and behaviour. One such event, ‘climategate’, involved leaked emails which appeared to show poor scientific practice amongst climate science researchers (Collins, 2014, pp. 11–14; John, 2018). The result was a public backlash that empowered climate change sceptics, who depicted the behaviour of the scientists involved as typical of climate science more generally, and further claimed that this showed a conspiracy to mislead people about the dangers of climate change. But this was not a voluntary airing of flaws in research practices, and needn’t reflect what would be expected if the medical actors *themselves* made BMU more widely known.

Another area of relevance is research into how people respond to uncertainty, and how this relates to trust. The evidence regarding responses to scientific and medical uncertainty is mixed. It is well established that people find the *experience* of uncertainty (in the form of ambiguity—the psychological state of being uncertain) to be aversive (Keren & Gerritsen, 1999). Yet a state of ambiguity need not follow from receiving information regarding uncertainty. One might be told that the probability of a drug improving one’s chronic pain is anywhere between 10 and 70% and be quite certain that one wishes to receive the medication. Further, a dislike of ambiguity need not deter us from the provision of information likely to provoke uncertainty, since there may be more at stake than experiencing some unpleasantness in the short term.

Research looking specifically at how informational uncertainty interacts with trust and other outcomes of interest is somewhat conflicting. Some evidence suggests that providing uncertain information regarding health risks (for instance, through a range estimate of those risks) may variously reduce understanding, increase risk perception, decrease the credibility of the information provider, and decrease patient decision satisfaction (Longman et al., 2012; Politi et al., 2010). Others speculate that uncertainty regarding environmental safety can make people less trusting of government interventions and less willing to take environmental action (Johnson & Scicchitano, 2000). However, other research indicates that highlighting uncertainty in environmental/health risks *does not* increase risk perception nor reduce trust, and may increase people’s ratings of the current status of scientific knowledge (Wiedemann et al., 2006). Van der Bles et al. describe how “it is commonly assumed that communicating uncertainty transparently will invite criticism, can signal incompetence, or even decrease public trust in science” (Van Der Bles et al., 2020, p. 1). Yet, in a number of studies, they found little evidence to support this, finding that uncertainty produced only a small decrease in trust in ‘the numbers’ and in the information source.

It is quite possible that a controlled, voluntary move towards greater openness and acknowledgement of BMU could harbour trust, rather than erode it. However, as Van der Bles and colleagues point out, even if communicating uncertainty does lead to some reduction in trust, this might be appropriate if confidence in ‘the numbers’ was previously unreasonably high. This brings us to (ii): even if communication of uncertainty does diminish trust (and perhaps also decreases decision satisfaction, patient experience, or has other ‘negative’ effects), would this justify practices which disguise BMU and foster overoptimism about the effectiveness of many medical interventions?

As argued above, there are epistemic, professional and instrumental reasons that point towards an ethical presumption in favour of greater openness and a Corrective Response. Such an assumption, however, has been challenged, for instance by John (2018) who argues that:

Unfortunately, just as publicising the inner workings of sausage factories does not necessarily promote sausage sales, so, too, transparency about knowledge production does not necessarily promote the flow of true belief throughout the population (and so on for honesty, sincerity and openness)

(John, 2018, p. 75).

John argues that demands for scientists to be transparent (as well as sincere, open and honest)^7^ can actively obstruct the promotion of true beliefs regarding scientific claims amongst lay folk.

There are a number of ways in which those advocating for a Corrective Response might respond to or incorporate aspects of John’s account. First, John points to climategate as a “massive experiment in transparency” (John, 2018, p. 75) which reveals that lay people’s ‘folk philosophy of science’ does not permit the kinds of practices that are commonplace amongst climate science (and presumably other) researchers to count as good scientific practice. ‘Transparency’ here (again, not deliberately engaged in, but ‘forced’ through leaked information, possibly taken out of appropriate context) only served to undermine trust. Yet, we might still think that norms such as (intentional) transparency and openness have some value, though require nuance in their application. A more considered release of information than was permitted in the climategate example, with more opportunity to take care with *which* and *how* details of scientific practices are communicated, would be necessary. Just as bravery ceases to be a virtue when it involves a reckless and unnecessary disregard for one’s safety, the manner in which transparency and openness are enacted will determine whether behaviours appropriately display those virtues.

It may be the case that different areas of science require different norms. The example used by John, climate science, is a highly contested and politically polarised area of urgent concern. Some of the instrumentalism John thinks should be permitted here (in order to achieve valuable ends such as greater commitments to combatting climate change) might not be necessary or appropriate in other areas of science. If so, climate science might not provide a particularly helpful model for deciding what our norms of scientific communication ought to look like in general.

Some areas of medicine, however, might share similar features to the climate science case: debates around the safety of COVID-19 vaccines and mask use involve an urgent, politically charged area, where scientific uncertainty and expert disagreement risk undermining public trust and cooperation (Remmel, 2021).^8^ Indeed, public health leaders have been accused of instrumentalism in their comments regarding mask use and COVID-19 vaccination, by making claims about the (lack of) benefits of mask wearing in ways that are misleading, so as to protect supply (Powell & Prasad, 2021; Prasad, 2020). Perhaps this is another area where norms of openness need not apply.

Whilst it is at least *plausible* that a consequentialist case for deploying information instrumentally (and ignoring norms of openness) could be made, it is unclear how well placed we are, in general, to know that this is so. The backlash against the perceived instrumentalism of Anthony Fauci (as well as the UK government, the WHO and others) in statements regarding masking and vaccination for COVID-19 suggests a loss of trust that could be damaging in the long term (Tufekci, 2020). Furthermore, it may have been unnecessary: at least some research suggests that, for instance, providing uncertainty information regarding vaccine effectiveness does not change people’s intentions to vaccinate (Kerr et al., 2021).

Communication about the effectiveness of medical interventions, including about uncertainty and lack of good quality evidence, will require careful attention to the way in which those messages are interpreted, and how they interact with people’s folk philosophy of science. That is, successful communication must be sensitive to people’s model of how science should ‘work’: what they perceive as ‘good’, trustworthy science and what they recognise as corrupt and unreliable. In order to be in a position where more openness regarding the practices of medical research is feasible without causing harm, it might be necessary to put significant work into addressing people’s inaccurate folk philosophies of science, where these are likely to lead to misinterpretations of scientific evidence. For instance, some research suggests health literacy can be improved by teaching evidence based medicine as part of school curricula (Chalmers et al., 2018; Nsangi et al., 2017; Steckelberg et al., 2009). Other tools and decision aids (such as fact boxes or pictorial representations) might be used in order to better equip people to judge uncertain medical information they encounter (Gigerenzer et al., 2007; Smith et al., 2010). Given the emphasis within medical ethics placed on patient involvement in decision making and the (proclaimed) importance of ensuring patients are properly informed about prospective treatments, it would be worthwhile investing in a future where people are aware of BMU and able to judge the relevance of medical information they are given.

## Concluding remarks

In this paper, we have described the features of Broad Medical Uncertainty and the ways in which medical research and communication practices contribute to it. These practices not only leave us with significant and underestimated uncertainty regarding the effects of medical interventions, but lead to systematic overoptimism about the benefits and harms of treatments.

BMU doesn’t imply a thoroughgoing scepticism of medicine in general. Fortunately, there are medical interventions where the benefits are so large as to render uncertainty, in practice, unimportant. It would seem churlish to quibble over the exact effectiveness of the smallpox vaccine given its dramatic life-saving benefits. But for interventions where the benefits are less stark, the harms more uncertain, and the costs much greater, uncertainty can cloud the difference between an intervention being all-things-considered worth adopting and it being best avoided.

We have argued that there are ethical reasons based on epistemic, professional, and instrumental considerations that warrant a presumption for openness regarding BMU and suggest that a Corrective Response should be adopted. Whilst we have not described in detail what this would involve, it would centrally require active efforts to better inform people about the presence of BMU and the likely overestimate of treatment benefits (and underestimate of harms) embedded in the medical research upon which they and their medical team must base treatment decisions. As indicated, there is a significant literature on health communication, as well as research on how to promote health literacy and statistical understanding amongst both patients and doctors, which can guide efforts to inform people about BMU. We have focused on objections to the argument that a Corrective Response is warranted, including the risk that it will cause people to under-engage with medical treatments, and that it could provoke a damaging loss of trust in medical and scientific institutions in general. We do not consider there is sufficient evidence to support the claim that a Corrective Response would result in such harms so as to uphold the Anti-Corrective Responses we discuss. Absent further competing evidence to uphold the Anti-Corrective Response, we therefore consider there to be an ethical obligation to better inform people about the pervasiveness of BMU.

## Data Availability

Not applicable.

## Footnotes

1 We use the term ‘overly optimistic’ to refer to the inflated expectations people have about the effectiveness of medical interventions. We consider *overly* optimistic (rather than simply optimistic) to be appropriate where expectations of treatment benefits are not justified on the basis of the evidence (and meta-evidence) available. This is not to say there is something *normatively* mistaken about adopting an attitude of optimism.

2 It is known that clinicians’ expectations are optimistic relative to trial data (Hoffman & Del Mar, 2017), and as we describe, trial data are likely to also be optimistic.

3 We might further think of treatments which have very high rates of effectiveness—say, 99% of recipients experience a clear net benefit. These will be rare, creating *ultra*-narrow medical uncertainty or even medical certainty.

4 In a deterministic universe aleatory uncertainty may be reducible to epistemic uncertainty. Whether or not we live in a deterministic universe, in practice, certain details about the future are not predictable and for all intents and purposes can be considered aleatory.

5 O’Neil specifies that information must be made accessible, intelligible, assessable and useable.

6 Thanks to an anonymous reviewer for this suggestion.

7 John does not define transparency and openness, but treats the two as sufficiently similar to group together. As discussed we have used the term openness, to reflect not only the possibility of sharing additional information with patients and the public regarding treatment effects, but also to reference a move towards ‘open science’ and more active engagement of patients with medical research findings.

8 Thanks to an anonymous reviewer for highlighting this case.
